# Associations between the acclimation of phloem-cell wall ingrowths in minor veins and maximal photosynthesis rate

**DOI:** 10.3389/fpls.2014.00024

**Published:** 2014-02-06

**Authors:** William W. Adams III, Christopher M. Cohu, Véronique Amiard, Barbara Demmig-Adams

**Affiliations:** ^1^Department of Ecology and Evolutionary Biology, University of ColoradoBoulder, CO, USA; ^2^Genomics and Bioinformatics Unit, Agriaquaculture Nutritional Genomic CenterTemuco, Chile

**Keywords:** biotic defense, companion cells, light acclimation, leaf vasculature, phloem, photosynthesis, temperature acclimation, transfer cells

## Abstract

The companion cells (CCs) and/or phloem parenchyma cells (PCs) in foliar minor veins of some species exhibit invaginations that are amplified when plants develop in high light (HL) compared to low light (LL). Leaves of plants that develop under HL also exhibit greater maximal rates of photosynthesis compared to those that develop under LL, suggesting that the increased membrane area of CCs and PCs of HL-acclimated leaves may provide for greater levels of transport proteins facilitating enhanced sugar export. Furthermore, the degree of wall invagination in PCs (*Arabidopsis thaliana*) or CCs (pea) of fully expanded LL-acclimated leaves increased to the same level as that present in HL-acclimated leaves 7 days following transfer to HL, and maximal photosynthesis rates of transferred leaves of both species likewise increased to the same level as in HL-acclimated leaves. In contrast, transfer of *Senecio vulgaris *from LL to HL resulted in increased wall invagination in CCs, but not PCs, and such leaves furthermore exhibited only partial upregulation of photosynthetic capacity following LL to HL transfer. Moreover, a significant linear relationship existed between the level of cell wall ingrowths and maximal photosynthesis rates across all three species and growth light regimes. A positive linear relationship between these two parameters was also present for two ecotypes (Sweden, Italy) of the winter annual *A. thaliana* in response to growth at different temperatures, with significantly greater levels of PC wall ingrowths and higher rates of photosynthesis in leaves that developed at cooler versus warmer temperatures. Treatment of LL-acclimated plants with the stress hormone methyl jasmonate also resulted in increased levels of wall ingrowths in PCs of *A. thaliana* and *S. vulgaris* but not in CCs of pea and *S. vulgaris*. The possible role of PC wall ingrowths in sugar export versus as physical barriers to the movement of pathogens warrants further attention.

## INTRODUCTION

Transfer cells, specialized for facilitating movement of molecules or ions into or out of cells as a result of enhanced cell-membrane area into which transport proteins are embedded, can be found at key junctures within and between plant tissues (Offler et al., 2003). The increased cell membrane area is achieved through invagination of the cell wall, leading to a labyrinth of wall and plasma membrane. In seeds of cotton, greater cell-wall ingrowths have been associated with a higher yield of fiber and seed biomass (Pugh et al., 2010), presumably due to greater fluxes of reduced carbon and nutrients from the mother plant to the developing seed. On the other hand, seed development was inhibited in a pea line with a mutation that blocked development of transfer cells between mother plant and seed (Borisjuk et al., 2002). There is thus clear evidence for a role of transfer cells in increasing the flux of nutrients into sinks resulting in enhanced sink development.

Transfer cells also occur in source tissues, e.g., in the phloem of minor leaf veins (Gunning et al., 1968; Offler et al., 2003). Such cells show enhanced levels of invagination in response to various environmental conditions, including growth at higher levels of light (Henry and Steer, 1980; Wimmers and Turgeon, 1991) and, for the winter annual *Arabidopsis thaliana*, growth at lower temperatures (Edwards et al., 2010). Leaves of most plants grown in high compared to low light (Amiard et al., 2005), as well as those of winter annuals grown at low compared to warm temperature (Adams et al., 2013; Cohu et al., 2013b, 2014), exhibit higher rates of light- and CO_2_-saturated photosynthesis, and may therefore have a higher capacity for foliar sugar export. The extent to which transfer-cell enhancements in minor veins may enable increased levels of source activity has received little attention, although greater levels of sucrose export were associated with higher levels of wall ingrowth in pea (Wimmers and Turgeon, 1991). In addition, it was noted that pea leaves grown under high versus low light had both higher maximal rates of photosynthesis and higher levels of companion cell wall ingrowths (Amiard et al., 2005). Furthermore, several species exhibited greater levels of wall ingrowth in either phloem parenchyma or companion cells (or both cell types) in leaves grown under high versus low light (Amiard et al., 2007).

In the present study, a comprehensive examination of associations between the extent of foliar minor-vein transfer-cell wall ingrowth and photosynthetic capacity was undertaken for leaves of three species grown under three different growth light regimes and for the leaves of two ecotypes of *A. thaliana* grown under three different temperature regimes. In addition, the impact of the stress hormone methyl jasmonate on foliar phloem-cell wall ingrowths is also reviewed, calling for a resolution (by future research) of the question of an involvement of phloem-parenchyma cell-wall ingrowth in either sugar export (as suggested by correlations between ingrowth level and maximal photosynthesis rate) and/or potential physical barriers to the passage of pathogens (as suggested by the responsiveness to methyl jasmonate).

## MATERIALS AND METHODS

### PLANTS, GROWTH CONDITIONS, AND EXPERIMENTAL TREATMENTS

Three species with transfer cells in their foliar minor veins (Pate and Gunning, 1969; Henry and Steer, 1980; Haritatos et al., 2000), *A. thaliana* (L.) Heynhold (ecotype Columbia, i.e., Col-0), *Pisum sativum* L. cv. Alaska (pea), and *Senecio vulgaris* L., were grown as described in Amiard et al. (2007) under either 100 μmol photons m^-^^2^s^-^^1^ (low light = LL) or 1000 μmol photons m^-^^2^s^-^^1^ (high light = HL) at 25°C during the day and 20°C during the night. A third group of plants were grown under LL-conditions and then abruptly transferred to HL-conditions for a period of seven days. A fourth and fifth group of plants were grown under LL-conditions and sprayed daily with a solution of either 10 μM methyl jasmonate (MeJA) in water and 0.05% Tween or only water and 0.05% Tween (control group for the MeJA-treated group) for 7 days. To evaluate the impact of growth temperature on transfer-cell wall ingrowths and photosynthesis, two ecotypes of *A. thaliana* (from Sweden and Italy; see Ågren and Schemske, 2012) were grown as described in Cohu et al. (2013a) under 400 μmol photons m^-^^2^s^-^^1^ with day/night leaf temperatures of approximately 14/12.5°C, 18/15°C, or 36/25°C. Only mature leaves that had expanded fully under the respective light and temperature growth conditions were characterized, with the exception that leaves transferred from LL to HL, or sprayed with a solution, had expanded fully prior to the 1-week treatment in each case.

### MINOR-VEIN TRANSFER CELL CHARACTERIZATION

Leaf tissue was fixed, sectioned, and stained, and electron micrographs of minor veins imaged, as described in Amiard et al. (2005). The percentage increase in cross-sectional cell membrane perimeter, relative to such cells possessing no cell wall ingrowths, was determined according to Wimmers and Turgeon (1991) as described in Amiard et al. (2005) using EEB Viewer software or Image-J (Rasband W. S., ImageJ, U.S. National Institute of Health, Bethesda, MD, USA, http://imagej.nih.gove/ij/, 1997-2012).

### PHOTOSYNTHETIC CAPACITY (MAXIMAL RATE OF PHOTOSYNTHESIS)

The rate of light- and CO_2_-saturated photosynthetic oxygen evolution at 25°C in a water-saturated atmosphere was determined from leaf disks (Delieu and Walker, 1981) as described in Amiard et al. (2005). Saturating levels of CO_2_ (5%) were provided to overcome all resistances (stomatal, cuticular, and mesophyll-related) to CO_2_ diffusion to the chloroplasts. Saturating light of 1,475 or 2,425 μmol photons m^-^^2^s^-^^1^ was used for leaves grown under 100 versus 400 and 1000 μmol photons m^-^^2^ s^-^^1^, respectively.

### STATISTICAL ANALYSES

For the Columbia ecotype of *A. thaliana* (Col-0), pea, and *S. vulgaris*, mean values of light- and CO_2_-saturated photosynthetic oxygen evolution (±standard deviation) were determined from three leaves from three different plants, and mean values for percent increase in plasma membrane length (±standard error) were determined from 26 to 87 cells from three to six leaves each. In the case of the Swedish and Italian ecotypes of *A. thaliana*, mean values of light- and CO_2_-saturated photosynthetic oxygen evolution (±standard deviation) were determined from three to four leaves from three to four different plants, and mean values for percent increase in plasma membrane length (±standard error) were determined from 27 to 48 cells from three to four leaves each. Comparison of means (ANOVA or *t*-test) and correlation coefficients and significance level of linear relationships (ANOVA) were determined using JMP software (SAS Institute, Cary, NC, USA).

## RESULTS

**FIGURE 1:**
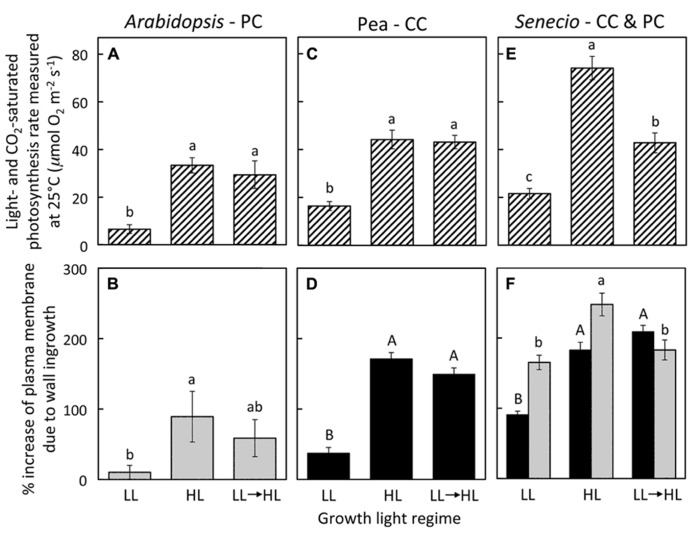
**(A,C,E)** Foliar light- and CO_2_-saturated rate of photosynthetic oxygen evolution ascertained at 25°C and **(B,D,F)** percent increase in plasma membrane length due to cell wall ingrowths estimated from cross-sections of phloem transfer cells (parenchyma = PC, companion = CC) relative to the hypothetical membrane area in the absence of wall ingrowths in foliar minor veins of Italian **(A,B)**, pea **(C,D)**, and *S. vulgaris* **(E,F)** that developed in low light (LL), high light (HL), or LL and then transferred to HL for seven days. Light-gray columns indicate PCs and black-filled columns indicate CCs. Significant differences between means (*P* < 0.05) within each species are denoted by different lower-case letters, with the exception that upper-case letters are used to designate significant differences among the level of cell wall ingrowths for CCs, i.e., means sharing a common letter are not statistically different from each other. Photosynthetic capacities for pea from Amiard et al. (2005) and for *A. thaliana* and *S. vulgaris* grown in HL from Amiard et al. (2007).

**FIGURE 2:**
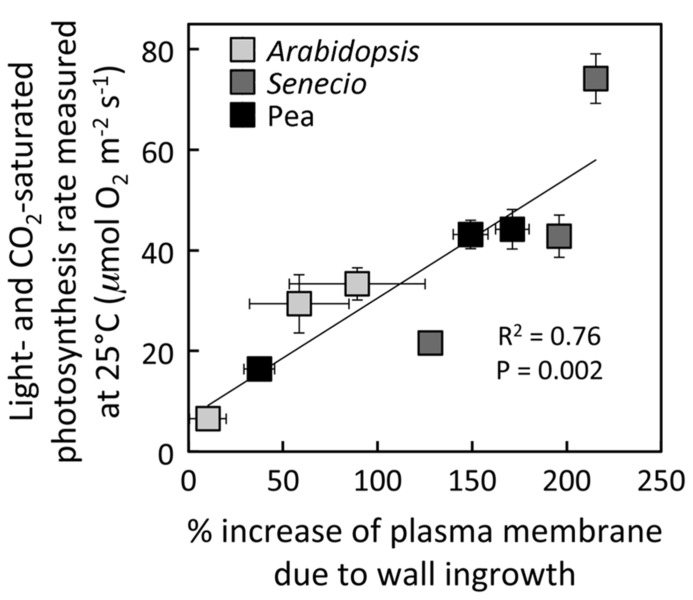
**Relationship between the percent increase of transfer-cell plasma membrane length in foliar minor veins and photosynthetic capacity from leaves of *A. thaliana* (light gray), pea (black), and *S. vulgaris* (dark gray) developed under LL or HL, or seven days after transfer of leaves developed under LL to HL.** For *S. vulgaris*, the values for percent increase in transfer-cell plasma membrane length are averages of the mean values obtained from PCs and CCs. For additional information, see **Figure 1**.

Growth of *A. thaliana*, pea, and *S. vulgaris* under HL resulted in leaves with significantly higher photosynthetic capacities (maximal rates of photosynthesis; **Figures 1A,C,D**) and foliar minor veins with phloem transfer cells possessing a significantly higher level of cell-wall ingrowths (**Figures 1B,D,F**) compared to leaves from plants grown under LL. Photosynthetic capacity of fully expanded leaves of *A. thaliana* and pea was not statistically different from (i.e., was equivalent to) the high rates of HL-acclimated leaves one week following transfer of LL-acclimated plants to HL (**Figures 1A,C**), and the extent of transfer-cell wall invagination of minor vein phloem was also not statistically different between HL-acclimated leaves and those transferred from LL to HL (**Figures 1B,D**). On the other hand, fully expanded leaves of *S. vulgaris* plants transferred from LL to HL exhibited only partial upregulation of photosynthesis that was not only significantly higher than that of LL-acclimated leaves, but also significantly lower than that of HL-acclimated leaves (**Figure 1E**). The extent of minor-vein companion cell (CC) wall ingrowths of LL-to-HL transferred *S. vulgaris* leaves was significantly greater than that of LL-acclimated leaves and equivalent to that of HL-acclimated leaves (**Figure 1F**). On the other hand, minor-vein parenchyma cell (PC) wall ingrowths of LL-to-HL transferred *S. vulgaris* leaves were the same as that of LL-acclimated leaves and therefore significantly lower than that of HL-acclimated leaves (**Figure 1F**). Plotting of the data from **Figure 1** revealed a positive linear relationship between the level of minor-vein phloem transfer-cell wall ingrowth and photosynthetic capacity among all three species and all three growth-light conditions (**Figure 2**).

**FIGURE 3:**
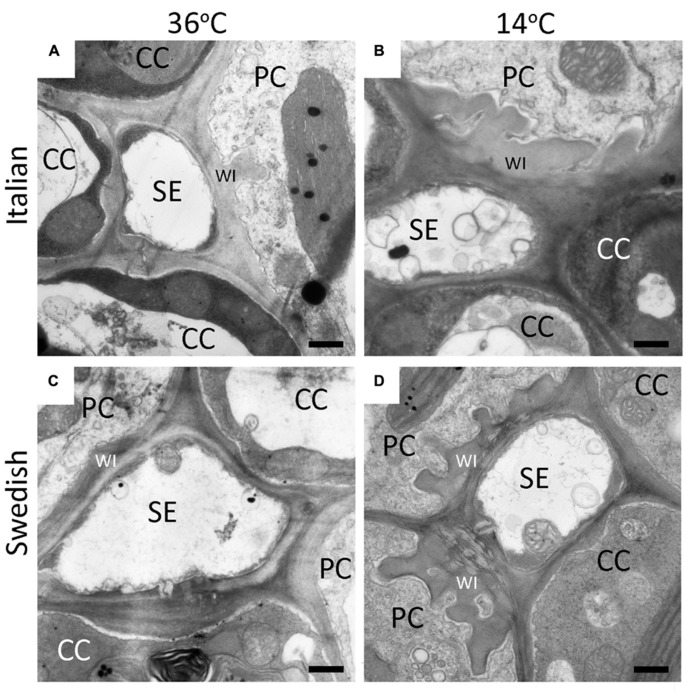
**Representative transmission electron-micrographic cross-sectional images of sieve elements (SE) surrounded by parenchyma (PC) and companion (CC) cells in foliar minor veins of Italian (A,B) or Swedish **(C,D)** ecotypes of *A. thaliana* that developed under 400 μmol photons m^**–**^^**2**^s^**–**^^**1**^ at a daytime leaf temperature of 36°C **(A,C)** or 14°C **(B,D)**.** Cell-wall ingrowths (WI) in the PCs are adjacent to the area abutting a neighboring SE, sometimes extend over the area of adjacent CCs (particularly apparent at lower temperature), and are more pronounced (greater level of invagination) in both ecotypes that developed at lower temperature. Black bar = 500 nm in length.

**FIGURE 4:**
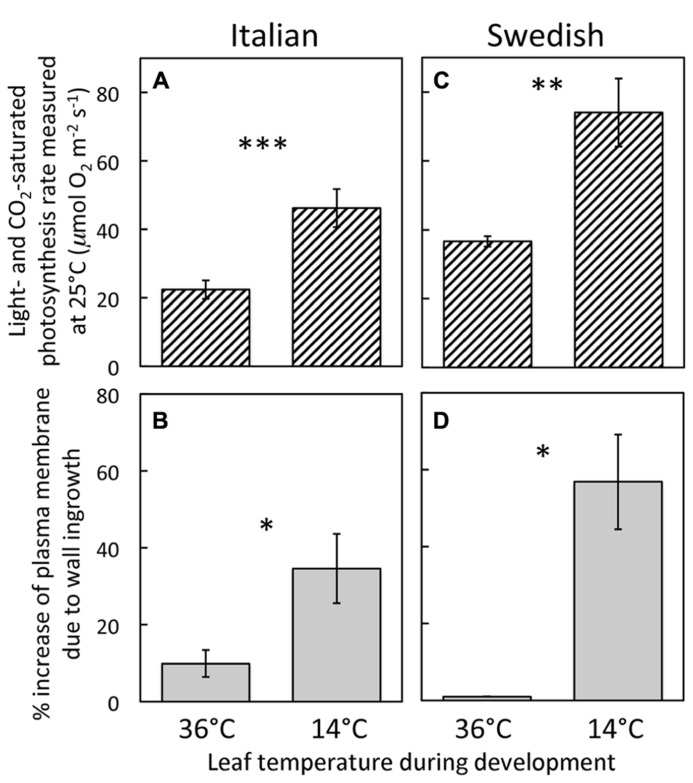
**(A,C)** Foliar light- and CO_2_-saturated rate of photosynthetic oxygen evolution ascertained at 25°C and **(B,D)** percent increase in plasma-membrane length due to cell-wall ingrowths estimated from cross-sections of phloem-parenchyma transfer cells relative to the hypothetical membrane area in the absence of wall ingrowths in foliar minor veins of Italian **(A,B)** and Swedish **(C,D)** ecotypes of *A. thaliana* that developed under 400 μmol photons m^-^^2^s^-^^1^ at daytime leaf temperatures of 36 or 14°C. Significant differences between the means within each ecotype are denoted by asterisks (*P < 0.05, **P < 0.01, and ***P < 0.001). Photosynthetic capacities from leaves that developed at 14°C from Cohu et al. (2013b).

**FIGURE 5:**
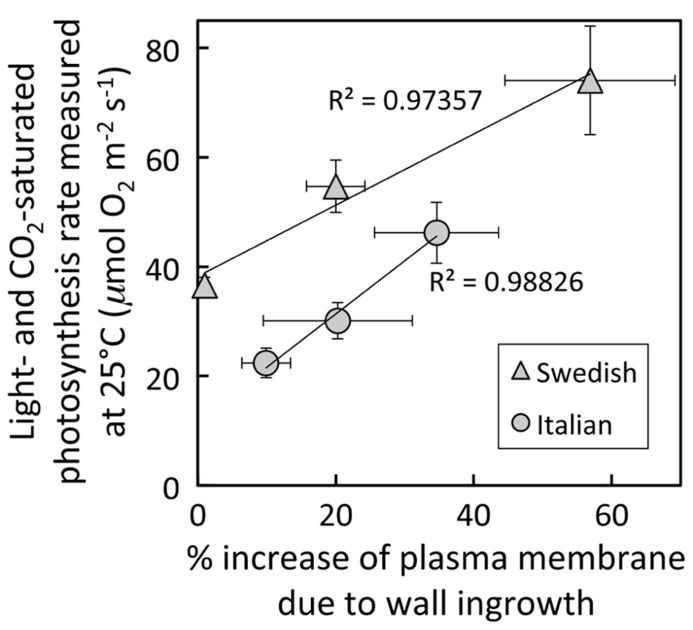
**Relationship between the percent increase of parenchyma-cell plasma-membrane length in foliar minor veins and photosynthetic capacity from leaves of Italian (circles) or Swedish (triangles) ecotypes of *A. thaliana* that developed under 400 μmol photons m^**–**^^**2**^s^**–**^^**1**^ at a daytime leaf temperature of 36°C (lowest values), 18°C (intermediate values), or 14°C (highest values).** The linear relationship among all six values (not shown) was significant at *P* <0.05, whereas the linear relationships shown for each ecotype were not. Photosynthetic capacities from leaves that developed at 14°C from Cohu et al. (2013b).

Development of leaves at low temperature (**Figures 3B,D**) resulted in enhanced levels of minor-vein phloem-PC wall ingrowths and photosynthetic capacities in both Italian (**Figures 3A,B**) and Swedish (**Figures 3C,D**) ecotypes of *A. thaliana* compared to development at warmer temperature (**Figures 3A,C**), such that both parameters were significantly higher in leaves of both ecotypes that developed at a leaf temperature of 14°C compared to leaves that developed at 36°C (**Figure 4**). Moreover, there were linear relationships between the level of such foliar minor-vein PC wall ingrowths and photosynthetic capacity of the leaves for each ecotype across three growth temperatures, with both metrics increasing significantly between growth at warm versus low temperature (**Figure 5**).

**FIGURE 6:**
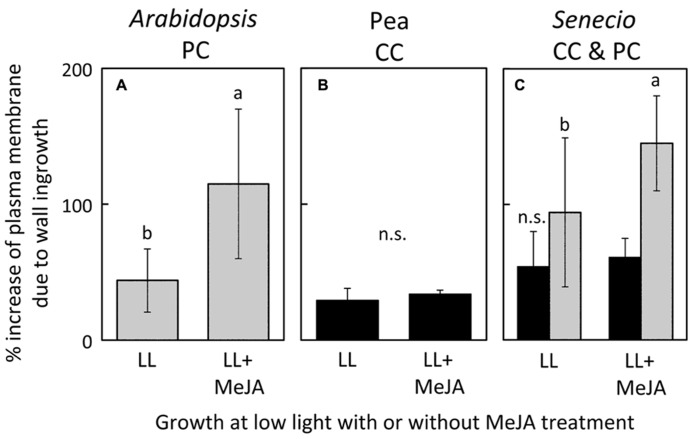
**Percent increase in plasma-membrane length due to cell-wall ingrowths estimated from cross-sections of phloem transfer cells (parenchyma = PC, companion = CC) relative to the hypothetical membrane area in the absence of wall ingrowths in foliar minor veins of *A. thaliana***(A)**, pea **(B)**, and *S. vulgaris***(C)** that developed in low light and were sprayed with a 0.05% solution of Tween (LL) or a 0.05% solution of Tween with 10 μM methyl jasmonate (LL+MeJA) for seven days. Gray columns indicate PCs and black columns indicate CCs.** Significant differences between the means (*P* <0.05) of *A. thaliana* **(A)** and *S. vulgaris* **(C)** are denoted by different lower case letters. Percent increase of plasma membrane length was not significantly different (n.s.) between control and MeJA-treated companion cells of either pea **(B)** or *S. vulgaris* **(C)**. Data from Amiard et al. (2007).

Treatment of LL-acclimated leaves with methyl jasmonate for one week resulted in a significantly greater level of cell wall ingrowths in the foliar minor vein phloem PCs of both *A. thaliana* (**Figure 6A**) and *S. vulgaris* (**Figure 6C**), but had no impact on the level of cell wall ingrowths in minor-vein phloem CCs of either pea (**Figure 6B**) or *S. vulgaris* (**Figure 6C**).

## DISCUSSION

The regulatory factors modulating deposition of cellulose and lignin to form wall ingrowths are a subject of active research (e.g., Edwards et al., 2010; Arun Chinnappa et al., 2013). It has recently been demonstrated that reactive-oxygen species play a central role in stimulating such ingrowth formation (Andriunas et al., 2012), as we had postulated previously (Amiard et al., 2007). Any abiotic stress, such as the high growth light or low growth temperatures employed in this study, might be expected to generate higher levels of reactive oxygen species (Suzuki et al., 2012). Wounding through attack by pests or infection by pathogens also leads to elevated levels of reactive oxygen (Bell et al., 1995; Torres, 2010). Increased levels of reactive oxygen can, in turn, be expected to result in increased formation of oxylipin hormones (e.g., jasmonic acid and methyl jasmonate) through peroxidation of polyunsaturated fatty acids (Demmig-Adams et al., 2013). An increase in the synthesis of jasmonate hormones, that stimulate jasmonate responsive genes important to the defense of plants, is one of the major responses of plants to abiotic and biotic (e.g., pathogen and insect attack) stress (Avanci et al., 2010; Ballare, 2011; Kazan and Manners, 2011; Hu et al., 2013; Santino et al., 2013).

The synthesis of jasmonates has, moreover, been localized to the vasculature of plants (Hause et al., 2003; Stenzel et al., 2003; Howe, 2004). This is perhaps not surprising, given that the PCs are typically the primary cells of the phloem subject to invasion by pathogenic viruses and fungi (Ding et al., 1995, 1998; Heller and Gierth, 2001; Zhou et al., 2002) as a means to gain access to sieve elements for distribution throughout the plant (Gilbertson and Lucas, 1996; Lucas and Wolf, 1999; Waigman et al., 2004; Scholthof, 2005; Vuorinen et al., 2011). The specific localization of PC-cell wall ingrowths to the region adjacent to sieve elements, coupled with the fact that PC wall ingrowth development was stimulated by MeJA, are both consistent with the hypothesis that such augmentation of cell wall material could serve as an increased physical barrier to the transmission of pathogens into the plant vascular system (for further discussion, see Amiard et al., 2007 and Demmig-Adams et al., 2013). Furthermore, the greater level of cell-wall ingrowth in the Italian compared to the Swedish *A. thaliana* ecotype when grown at higher temperature (36°C) may be related to the higher temperatures naturally experienced by the Italian ecotype (Ågren and Schemske, 2012) as well as the greater susceptibility to pathogenic infection that plants may experience at elevated temperature (Celebi-Toprak et al., 2003; Qu et al., 2005).

While higher light and lower temperature both represent conditions of potentially greater levels of excess light and therefore the potential for higher levels of reactive oxygen formation, they also represent the opportunity for higher rates of photosynthesis. The greater energy content of a higher flux of photons can be beneficial for any species capable of upregulating photosynthesis in the absence of environmental and/or genetic constraints (Amiard et al., 2005), and winter annual species likewise respond to lower temperature with strong upregulation of photosynthesis (Cohu et al., 2013b, 2014). The significant correlation between the level of minor-vein phloem cell wall ingrowths and photosynthetic capacity in the leaves of multiple species/ecotypes grown under different conditions of light and temperature documented here would be consistent with a role of these transfer cells in supporting foliar export of the products of photosynthesis. The increase in plasma membrane resulting from such ingrowths may thus serve to facilitate greater levels of assimilate export through (1) an increased area for sugar efflux from PCs into the apoplast adjacent to the sieve elements and CCs or from the apoplast to the cytosol of the CC transfer cells, (2) increased levels of ATPases for actively transporting protons from the cytosol of the transfer cells to the apoplast, and (3) increased levels of H^+^-sucrose symporters for moving sucrose from the apoplast into CC transfer cells as a critical step in the active loading of the phloem (Pate and Gunning, 1972; Gunning and Pate, 1974; Kühn, 2003; Offler et al., 2003; Sondergaard et al., 2004; Amiard et al., 2007). Cell-wall ingrowths provide a scaffold on which to lay down a significantly higher plasma-membrane area in which greater numbers of membrane-spanning transport proteins can be embedded. It should be kept in mind, however, that the metric assessed in the present study, as a two-dimensional measure of cell membrane length from cross-sections of transfer cells, can only serve as a proxy for the actual magnified membrane area arising from the labyrinth of cell-wall ingrowths.

It has been firmly established that the level of demand for products of photosynthesis by distant sinks in the plant can influence the rate of photosynthesis in source leaves, e.g., higher levels of utilization of sugars through metabolism, growth, and/or storage by heterotrophic, non-photosynthetic tissues result in higher rates of photosynthesis (Paul and Foyer, 2001; Körner, 2013). However, recent evidence indicates that features influencing the flux of reduced carbohydrates between photosynthetic mesophyll cells of the leaf and the plant’s sinks may also play a role in setting the upper ceiling for photosynthesis (Amiard et al., 2005; Adams et al., 2007, 2013; Cohu et al., 2013b, 2014; Muller et al., 2014; see also Ainsworth and Bush, 2011). Thus, despite the association between the degree of foliar minor-vein phloem cell-wall ingrowth and photosynthesis demonstrated in the present study, this relationship is only correlative in nature and should be considered as one of a suite of features that may be co-regulated and contribute to facilitating a given maximal rate of photosynthesis. For instance, in the case of *A. thaliana* (three ecotypes grown under four different sets of environmental conditions), we recently showed (Cohu et al., 2013b) that photosynthetic capacity is significantly correlated with number and cross-sectional area of minor-vein phloem cells (sieve elements, as well as CCs + PCs). In fact, the higher rate of photosynthesis for a given level of foliar minor-vein PC wall ingrowth in the Swedish compared to the Italian ecotype of *A. thaliana* shown in the present study may be related to higher numbers and a greater cross-sectional area of minor vein phloem cells in leaves of the Swedish ecotype relative to the Italian ecotype (Cohu et al., 2013a,b). Furthermore, across multiple species that load foliar veins apoplastically, photosynthetic capacity was significantly correlated with the product of foliar vein density and the number of phloem cells (Adams et al., 2013; Cohu et al., 2014; Muller et al., 2014). In addition, symplastic loaders do not have minor-vein phloem transfer cells, nor are transfer cells even present in all apoplastic loaders. Thus the degree to which minor-vein transfer cell walls are invaginated is an attractive candidate for being a contributor to the maximal photosynthesis that a leaf can exhibit, but is only one factor among many including, e.g., the number of palisade cell layers, vein density, characteristics of the xylem supplying water to the leaves, and other features of the phloem (Amiard et al., 2005; Dumlao et al., 2012; Cohu et al., 2013b, Cohu et al., 2014; Muller et al., 2014). The correlations between photosynthesis rate and phloem-cell wall invagination presented here clearly warrant further inquiry into a role of both CC and PC wall invagination in sugar export. Especially for the case of PC wall invaginations, the contrasting possible roles in sugar export versus obstruction of pathogen spread should be elucidated.

## Conflict of Interest Statement

The authors declare that the research was conducted in the absence of any commercial or financial relationships that could be construed as a potential conflict of interest.
